# Aggressive Behavior and Psychiatric Inpatients: a Narrative Review of the Literature with a Focus on the European Experience

**DOI:** 10.1007/s11920-021-01233-z

**Published:** 2021-04-07

**Authors:** Rosangela Caruso, Fabio Antenora, Michelle Riba, Martino Belvederi Murri, Bruno Biancosino, Luigi Zerbinati, Luigi Grassi

**Affiliations:** 1 Institute of Psychiatry, Department of Neurosciences and Rehabilitation, University of Ferrara, Ferrara, Italy; 2 University Hospital Psychiatry Unit and Consultation-Liaison Psychiatry and Program on Psycho-Oncology and Psychiatry in Palliative Care, S. Anna University Hospital and Health Trust, Via Fossato di Mortara 64a, 44121 Ferrara, Italy; 3 Department of Psychiatry, University of Michigan, Ann Arbor, MI USA; 4 University of Michigan Comprehensive Depression Center, Ann Arbor, MI USA; 5 Psycho-oncology Program, University of Michigan Rogel Cancer Center, Ann Arbor, MI USA; 6 Department of Psycho-oncology, Cancer and Molecular Medicine, University of Leicester, Leicester, UK; 7 Department of Mental Health, Ferrara, Italy

**Keywords:** Psychiatric inpatients, Aggressive behavior,, General hospital

## Abstract

**Purpose of Review:**

We summarized peer-reviewed literature on aggressive episodes perpetrated by adult patients admitted to general hospital units, especially psychiatry or emergency services. We examined the main factors associated with aggressive behaviors in the hospital setting, with a special focus on the European experience.

**Recent Findings:**

A number of variables, including individual, historical, and contextual variables, are significant risk factors for aggression among hospitalized people. Drug abuse can be considered a trans-dimensional variable which deserves particular attention.

**Summary:**

Although mental health disorders represent a significant component in the risk of aggression, there are many factors including drug abuse, past history of physically aggressive behavior, childhood abuse, social and cultural patterns, relational factors, and contextual variables that can increase the risk of overt aggressive behavior in the general hospital. This review highlights the need to undertake initiatives aimed to enhance understanding, prevention, and management of violence in general hospital settings across Europe.

## Introduction

While the trend of 1980s medical literature was to indicate the substantial lack of an increased risk for aggression in patients with psychiatric disorders [1], in the following decades, changes in mental health services including the development of consultation-liaison psychiatric units [2] have determined a different approach to this problem, and by the 1990s, the association between mental illness and aggression seemed to be confirmed [3–5].

Currently, findings suggest that up to 50% of patients with psychiatric disorders show aggressive symptoms, as compared to less than 2% of the general population [6•]. Likewise, recent studies hint at an increased risk of aggression among patients affected by mental disorders admitted to the hospital [7••, 8].

In Europe, significant levels of violence from patients in healthcare settings have been identified, with variance of reporting between countries, the highest rates being for France, UK, and Germany and the lowest rates for Norway and the Netherlands [9].

However, violence in healthcare settings is on the rise [10], to the point that the European Agency for Safety and Health at Work has defined the healthcare sector as one with the highest rates of violence [11]. The magnitude of the problems is such that a group of experts founded the “European Violence in Psychiatry Research group” (www.eviprg.eu) aimed to enhance the understanding, prevention, and management of violence in medical settings.

The aim of the present narrative review is to summarize recent peer-reviewed literature from European countries on aggressive episodes in adult patients with mental illnesses admitted to psychiatric units or general hospitals.

For this purpose, we will use the terms “aggression” and “violence” as synonyms and in accordance with the following definition: “physical, psychological, sexual, or other forms of actions which risk causing harm or paining the person exposed” [12].

## Aggression in Psychiatric Settings

Research into aggression in psychiatric services is fraught with difficulties. Many differences in methodology, in the definition of what is to be considered an aggressive behavior, as well as in the organization of psychiatry settings, determine a great variation in reported rates of aggression and make it difficult to compare and generalize findings [13].

The dimension of the phenomenon appears however critical if a recent meta-analysis highlighted that psychiatric nurses have about three times the odds of physical assault from patients than nurses in nonpsychiatric settings [14].

According to a review [15••], in acute psychiatric units, between 24 and 80% of mental health workers experienced at least one aggression during their career: verbal assaults affected 46–78.6% of staff, threats 43–78.6%, and sexual harassment 9.5–37.2%.

Another meta-analysis [16] of studies carried out on acute psychiatric wards in high-income countries showed that the pooled proportion of patients who committed at least one act of physical aggression was as high as 17%.

The documented rates of different kinds of reported aggression vary between continents, for example, it was suggested that sexual harassment rates seem lower in European studies [17], while reports of physical assault are more frequent in Anglo regions and bullying more common in the Middle East [18••].

As far as Europe is concerned, an interesting cross-sectional, comparative study between UK and Sweden using the Arnetz Questionnaire [19] found that, in psychiatric inpatient settings, more British than Swedish professionals were at risk of any kind (the study did not differentiate between verbal and physical aggression) of aggression by patients [20]. A more recent retrospective study, using the Patient Conflict Checklist [21] to analyze aggressive enactments in 522 patients of 84 acute psychiatric wards during the first 2 weeks of admission, showed that 51% of patients displayed verbal aggression in at least one occasion, 25% enacted aggression toward objects, and 20% was aggressive toward others. These findings suggest that in UK, aggression episodes might occur (or are reported) more frequently than in other European countries [22]. In general, however, in European hospital psychiatric settings, preoccupying rates of both verbal and physical violence are enacted by patients, and this condition has remained unvaried over the last two decades, according to recent studies based on specifically developed questionnaires and interviews carried out in Norway [23], England [24], and Italy [25].

A UK-based investigation among 194 subjects with a first episode psychosis using the Psychiatric and Personal History Schedule (PPHS) [26] showed that almost 40% of cases enacted an aggressive behavior and that approximately half of these were physically violent [27]. A very recent survey promoted by the UK National Health System [24] using ad hoc developed measures and existing instruments altered to fit the research intents found that the average number of aggressive incidents in mental health/learning disability trusts was over three times the average for all trusts.

According to a prospective study [28] exploring aggressiveness with the Overt Aggression Scale (OAS) [29], in nine French hospitals on involuntary psychiatric inpatients, 79% had been violent, either verbally or physically.

The situation seems comparable in Germany, where, according to a survey on four institutions, 78.7% of the interviewed professionals working in psychiatric inpatients settings reported physical aggression while 96.7% reported verbal forms of aggression from patients in the 12 months prior to the study [30].

Similarly, a Polish study [31] comparing acts of violence in psychiatric and nonpsychiatric settings showed that the overall level of aggression was significantly higher in psychiatric wards. Verbal aggression and screaming were experienced by 100% of psychiatric nurses and physical attacks by 79.5% during the previous year.

An Irish study [32] on patients with first episode psychosis using the Modified Overt Aggression Scale (MOAS) [33] found physical violence rates of 29% and verbal aggression rates of 36%.

A 1-year investigation on all patients admitted to two short-term psychiatric units in Norway, using the V-Risk-10 scale [34], revealed lower figures: 9% of 1017 patients were aggressive. Of the total number of episodes, 22% were verbal, 38% physical, and 40% both verbal and physical [35].

A Danish study, using the Brøset Violence Checklist (BVC) [36] as a predictor of both verbal and physical aggression risk in 15 psychiatric wards over a period of 3 months, registered very heterogeneous results, with risks for aggressive episodes varying from 0.1 (i.e., one expected violent episode for a patient observed in 1000 shifts) to 14.7% (147 expected violent episodes for a patient observed in 1000 shifts) [37].

In Italy, a questionnaire-based retrospective study [25] showed that 91.5% of the mental health professionals involved in the research reported that they had been victims of one or more episodes of aggressions in the previous year. Specifically, 90.8% reported verbal aggression, 44.5% physical aggressions, and 21.3% were victims of injuries.

The prevalence of aggression per patient was examined, using the Staff Observation Aggression Scale (SOAS) [38], in another Italian study of 1534 patients admitted to a psychiatric unit [39]. Of these, 116 resulted responsible for 329 aggressive episodes (prevalence of aggression = 7.5%; 2.8% incidents/patient). Thirteen episodes were verbal (4%), while the reminders were represented by physical assaults of different seriousness: 266 “simple” physical assaults (80.9%), 40 assaults by using objects 40 (12.1%), and 10 dangerous assault 10 (3%). These results were confirmed in a further 7-year follow-up study [40]. Similar findings were presented in a multicenter Italian study involving 1324 inpatients, of whom 10% showed a verbally hostile behavior or aggression toward objects and 3% were physically assaultive toward others [41].

## Aggression in Nonpsychiatric Settings

Interest in understanding how to deal with aggressive behavior in nonpsychiatric settings has increased over time.

International data on emergency departments (EDs) indicate that 25 to 80% of healthcare staff experience physical aggression at least once and nearly 100% is subjected to verbal aggression during their career [42]. Many factors contribute to aggression in EDs, such as poor staffing, lack of privacy, overcrowding, and availability of nonsecured equipment that can be used as weapons by patients [43••].

A review which undertook an international perspective [44] highlighted that modalities of aggression vary with different cultural and social contexts: for instance, patients presenting with weapons in the EDs are more frequent in countries where the acquisition of a weapon is legal, while it is much less likely in others, where obtaining a weapon is more difficult, such as the UK, where in contrast verbal threats, or intimidation, appear significant.

Violence in hospital is indeed considered critical in the UK, where National Health System statistics showed that there were almost one and a half million total reported assaults on staff in 2016 [45].

In Switzerland, a multiple regression analysis study evidenced that besides EDs, a high verbal and physical aggression hazard was present in intensive care units, recovery units, anesthesia, intermediate care, and step-down units [46]. A recent investigation carried out in Norway in ten emergency clinics over 1 year [47], using the Staff Observation of Aggression scale–Revised (SOAS-R), registered a total of 320 aggressive incidents, 60% of which were severe. Verbal aggression episodes accounted for 31.6% of all incidents, threats for 24.7%, and physical aggressions for 43.7%.

In geriatric settings, a Swedish study on 309 patients with dementia evidenced that nearly 32% of patients had shown a physical aggressive behavior in the preceding week. Violent patients were more likely to have impaired speech, difficulties in understanding verbal communication, and prescribed analgesics and antipsychotics [48]. In a German survey using SOAS-R, 73 and 94% out of 585 geriatric professionals had received respectively physical and verbal aggressions in the previous 12 months [49].

In an Italian national cross-sectional survey [50], 76% of 816 EDs nurses reported verbal abuse from patients, 15.5% both physical and verbal aggression, and 0.61% only physical abuse in the year prior to data collection. Rudeness and bad manners (94.0%), the threat of legal action (77.4%), and screaming or shouting (77.0%) were the most frequent verbal form of aggression. The most frequently reported physical aggressions were pulling/grabbing (50.4%), pushing (40.3%), and punching/slapping (36.4%). Arms (60.9%), chest (33.6%), and face (28.1%) were the most harmed parts of the body. Another Italian study, with a retrospective design, collected data from EDs and Acute Psychiatric Units staff, coming to similar findings [51], while a cross-sectional study on 700 nurses of several Italian health departments showed that 49% of them had experienced at least one episode of aggression in the previous year, 82% of which was verbal [52].

In a cross-sectional retrospective Polish study [31], 89.6% of nonpsychiatric nurses reported verbal aggression by patients in the previous year, while physical attacks were experienced by 20.6%. Thirty percent reported screaming from patients on a weekly basis, and over 16% repeated physical attacks. Similar findings leaded another extensive Polish study on more than 1600 healthcare workers, who declared both verbal and physical aggression by patients in several healthcare settings [53].

In general medical units, a condition known as excited delirium (ExD) frequently escalates to violent enactments [54]. ExD is a trans-diagnostic neuropsychiatric condition characterized by a cluster of behaviors including bizarreness, aggression, agitation, ranting, hyperactivity, paranoia, panic, surprising physical strength, hyperthermia, respiratory arrest, and not infrequently death [55]. It is the result of substance intoxication, psychiatric illness, alcohol withdrawal, head trauma, or a combination of these [56, 57].

## Risk Factors

The authors of a large longitudinal study carried out in Sweden found that subjects with mental disorders were approximately 4 times more likely than their healthy siblings to perpetrate violence [58••]. However, identifying the factors at the base of violent behaviors in this population is a difficult undertaking, given the wide range of potentially mediating variables.

For clarity reasons, we will divide the risk factors for aggression in psychiatric patients into “internal” and “external” factors [59•] (Table 1).

**Table 1:** The major risk factors for aggression in psychiatric settings

Internal factors	Clinical factors		Diagnosis of schizophrenia	Multiple combined factors
			Diagnosis of bipolar disorder	
			Acute psychotic symptoms	
			Mania	
	Premorbid conditions	Personal factors	Personality disorders	
			Impulsivity/hostility features	
			Younger age	
			Male gender	
			Poor insight	
		Historical factors	Past aggression	
			Past victimization	
			Psychopathy	
			Lower social class	
			Genetic factors	
			HPA axis	
			Functional/anatomic alterations	
	Substance misuse		Drug/alcohol abuse	
External factors	Environmental factors		Ward overcrowding	
			Day shifts	
	Admission variables		Involuntary admission	
			Higher length of stay	
	Relational factors		Ineffective interpersonal relationships	
			Lack of leadership	

Finally, we will account for recent research focusing on the neurobiological correlates of aggression [60, 61].

## Internal Factors

### Psychiatric Diagnosis

According to several studies [62–65], having a mental disease with severe symptoms is associated with a higher probability of any form of aggressive behavior. Recent investigations identify schizophrenia, cognitive impairment, anxiety, acute stress reaction, suicidal ideation, along with poor insight, personality disorders, impulsivity, and psychopathy as the clinical factors with the strongest empirical evidence for association with violence [66, 67]. In nonpsychiatric hospital setting, aggression, especially in a physical form, appears mostly perpetrated by patients affected by dementia, mental retardation, or other psychiatric disorders and by drug and substance abuse [68].

An extensive Swedish study using nationwide registers analyzed data on 250,419 psychiatric patients, showing that the risks of violence perpetration varied across diagnoses, with higher risks among patients with schizophrenia than among subjects with depressive disorders [58••]. However, according to another Swedish population-based longitudinal study, the risk of physical aggression was higher in subjects with depression than in the general population [69].

A Danish systematic study found an increased risk of aggressive acts associated with mental disorders along the entire diagnostic spectrum, with a similar effect size for schizophrenia, bipolar disorder, and depression [70].

Italian clinical studies showed a direct correlation between symptoms of acute psychosis and the occurrence of aggressive behaviors, with the presence of disorientation, motor hyperactivity, hostility-suspiciousness, grandiosity and borderline and passive-aggressive personality traits predicting a change for the worse in aggressive behavior, from verbal to physical [68, 71, 72••], while good metacognition and self-reflectivity appeared as protective factors [72••]. A recent retrospective Italian study found that clinical diagnoses with higher scores at the Modified Overt Aggression Scale (MOAS) were antisocial and borderline personality disorders, suicidality, mental retardation, and dementia [72••].

Another recent Italian cohort study [73] investigated the relationship between clinical and neuropsychological factors with risk of aggression in inpatients with schizophrenia. Physically aggressive patients showed a higher number of compulsory admissions, higher anger, and less negative symptoms as compared to non-aggressive patients. Aggressive patients performed better on executive and motor tasks probably due to lower negative psychotic symptoms. Negative symptoms also predicted fewer verbal aggressions at 1-year follow-up.

A recent Greek study suggested that a distinction should be done between aggressivity related to the core positive symptoms of schizophrenia and that emerging from independent antisocial personality traits, along with alcohol consumption [74].

Irish and UK observational studies [27, 32] evidenced that patients at first episode psychosis had an even higher risk of violent behavior when involuntarily treated, with a diagnosis of mania and the presence of manic symptoms.

An increased risk for aggressive behaviors has been associated with the concomitant presence of a complex psychiatric condition and the use of antiepileptic drugs in patients with epilepsy [75].

Recently, a role of both short sleep duration and night-to-night variations has been suggested in increased aggression among patients in psychiatric intensive care units [76].

### Sociodemographic Factors

Research, however, seems to suggest that mental disorders may not be necessary nor sufficient causes of violence and that, for psychiatric patients as for the general population, sociodemographic and economic factors are contributors to aggressive behaviors [16, 69, 72••, 77•, 78–82].

Male and younger patients are indicated as the most frequent perpetrators of aggressive enactments [66, 72, 83]. Nonetheless, a registry-based study carried out in Denmark showed that, while any form of aggression was much higher in men, the relative impact of mental disorders on risk of aggressive behaviors was stronger in women [69].

An Italian retrospective study [72••] on 400 patients evidenced that while mean MOAS scores were negatively correlated with age of patients, they resulted significantly higher in unemployed vs employed patients, as well as in patients living in indigence, in particular homeless. In addition, male and female single patients showed significantly higher mean MOAS scores compared to patients who were married, while no significant correlation with education and ethnicity was found.

These results are confirmed by a study which gathered data of 522 inpatients from 84 mental health units in South England. Physically aggressive patients typically were younger and more likely to live alone, indicating poor availability of social support [22].

A recent Czech clinical study [84] confirmed a negative correlation between employment and aggressiveness among patients with a psychotic disorder.

### History of Aggressions

Patients with severe mental disorders and a history of enacted aggressions are usually considered at high risk of recidivism and poor compliance [16].

A recent cohort Italian study [73] compared a sample of patients with schizophrenia and a history of physical aggression to patients with schizophrenia matched by age, gender, and alcohol abuse but without such a history. During a 1-year follow-up, patients with a history of aggression resulted more likely to be violent.

Another Italian prospective study, comparing never aggressive with verbally, physically, and sexually aggressive male psychiatric patients, found that the latter had displayed an increased number of aggressive behaviors in the previous 2 years [71].

On the same line, a UK study on 522 psychiatric inpatients evidenced that a history of violence was present at higher rates among those who enacted aggression of several forms during hospitalization [22]. However, in an Italian study, inpatients with a history of violence did not show at MOAS higher rates of aggressiveness compared to patients without such a history [85]. Interestingly, a second part of the study, exploring patients’ behavior in the community, found that subjects with a lifetime history of violence engaged in more aggressive enactments than patients without [86]. The Authors speculated that when adequate treatment and management are available, a history of physical aggressiveness does not link with an increased risk of violence.

### History of Childhood Abuse and Neglect

In psychiatric patients, as well as in the general population, aggressive conducts can be related to traumatizing life events, including childhood abuse and neglect [77•, 87].

The association between a history of childhood abuse and a higher risk of aggressive behavior and delinquency in the adult age (“the cycle of violence”) has been known for a long time [88], although empirical evidence emerged only more recently [89–92].

A cross-sectional UK study, comparing male mental health patients with a history of childhood trauma to matched patients without childhood traumatization, found that the formers were 2.8 more likely to commit physical aggressions across the life span [93].

A French study [87] evaluating five different forms of childhood and adolescence neglect and abuse in schizophrenic inpatients with a history of violence found that 46% experienced at least one form of abuse and/or neglect during childhood and 21% experienced more than two forms of abuse and/or neglect. The most frequent forms of maltreatment were physical abuse (39%) and emotional neglect (18%), followed by physical neglect (14%) and sexual and emotional abuse (11%). In addition, a large proportion of patients (43%) had lost a parent in their childhood, 42% of whom died from an aggression.

A retrospective study carried out in the Czech Republic [84] showed, using multiple regression, that childhood physical or sexual maltreatment and recent victimization robustly increased the risk of aggression in patients with psychosis.

Another recent Norwegian study [94] using the Childhood Trauma Questionnaire (CTQ) [95] found significant differences in childhood exposure to both abuse and neglect: aggressive psychiatric patients showed higher CTQ scores than non-aggressive patients, and both patient groups had higher scores than healthy controls.

### Drug Abuse

The role of substance abuse on aggressive enactments in psychiatry has been well articulated and described as one of the most robust clinical findings, with data indicating a doubling of the risk of physical violence through multiple pathways, including substances increasing psychotic symptoms, and a shared risk factors for psychosis and substance abuse [18••, 96••].

A recent review of 27 studies correlated substance abuse with the most severe form of aggressions in patients with schizophrenia [77•]. In a meta-analysis, moreover, substance abuse was associated with aggressions of any severity in patients at their first psychotic episode [97].

The impact of these findings is better understood when considering that, in Europe, 30–50% of people with a severe mental illness also have problems with substance use [98].

On this line, a recent Swiss cohort study on an early psychosis population of 265 patients found that the earlier the cannabis abuse started, the higher was the risk of physical aggressiveness [99].

An extensive registry-based longitudinal Swedish research, involving 250,419 patients, found that risk of aggression was the highest in subjects with concomitant substance use disorders [58••].

A population-based epidemiological study carried out in Denmark [69] confirmed that patients with comorbid mental illness and substance abuse increased the risk of physically aggressive enactments especially among women.

According to an abovementioned Italian study on psychiatric patients [72••], mean MOAS scores for both verbal and physical aggression resulted higher in the alcohol and cannabinoid abusers, while heroine abusers did not show increased scores.

Another study carried out in South England showed that illicit substance use was present in higher rates among verbally and physically aggressive inpatients, with no other clinical factors consistently related to inward aggressive behavior [22].

Alcohol intoxication, substance abuse, and mental illness were also indicated by 220 physicians and nurses as clinical factors for workplace violence in a Cypriot study [100],

Similarly, a retrospective Czech study estimated drug and alcohol use as predictors for physically assaultive behavior both in psychiatric inpatients and controls [84].

Cannabis abuse has been confirmed as a risk factor for violence in a Swiss investigation on patients with early psychosis. The combination of cannabis use, impulsivity, lack of insight, and non-adherence to treatment furtherly increased the risk [99].

A nationwide Italian research individuated among the major risks of physical aggression in EDs, drunkenness (44.6%), and being under the effect of illicit drugs (28.6%) [50].

## External Factors

### Contextual Factors

Starting from 1990s, literature has been uncontroversial in stating that interpersonal factors are among the most important external determinants of aggressive incidents in general hospital, with ineffective staff-patient relationships, aversive stimulations, and resulting feelings of powerlessness as dramatic precipitants of aggressive acting-outs [22, 67, 78, 101, 102].

A review specifically focused on staff-patient relationship [103] showed that a negative “staff-patient interaction” was the most frequent antecedent overall, precipitating 39% of all aggressive incidents. These finding were confirmed by a review on patients’ perception of factors that ignited a violent reaction toward the staff. Patients recognized in the experience of being ignored and in perception of staff behavior as custodial two major factors triggering aggressive reactions [104].

In Italy, high patient/nurse ratios have been indicated as another significant correlate of an increased risk of violence in general hospital [105], along with long waiting, overcrowding, and feeling of lack of caring [50].

Studies conducted on Norwegian and Irish staff individuated long waiting, involuntary examinations, and poor communication as major contributors to aggressive enactments in EDs [47, 106].

Conversely, a study conducted on English nurses indicated in a positive nurse-patient relationship, the most protective factor against risk of inward violence [107]. In addition, an interesting Italian research found that staff members’ avoidant attachment correlated positively with emotionally aggressive behaviors by patients, while a secure attachment style correlated negatively with both verbally and physically aggressive behaviors. [108•].

No “week-end effect,” in terms of significant variations in the incidence of aggressions on different days of the week due to changes in availability of personnel and services, was suggested in a large study using electronic health records of inpatients of a psychiatric hospital in England [109].

Regarding the physical structure of hospital wards, English and Swedish retrospective studies showed that in psychiatric wards with a distress reducing design (e.g., optimal light, privacy, common areas with ample spaces, and a garden), the number of physical restraints and compulsive injections was reduced [111, 110].

In recognizing the clinical environment as a factor that influences violent incidents, the Royal College of Psychiatrists in UK has developed guidelines to provide directions on the environmental and relational elements that trigger or reduce aggressive behaviors [112].

## Neurobiological Theories of Aggression

Studies on the neurobiology of aggression have proposed possible alterations in brain areas also involved with the formation of psychotic symptoms and affective regulation, including frontotemporal circuitry, the amygdala-orbitofrontal system, prefrontal cortex, and hippocampus [7, 113, 114••, 115••, 116].

Multiple lines of evidence have shown a dysfunction in the serotonin (5-HT) system in aggressive and in mentally ill subjects, with reduced 5-HT activity associated with depression as well as with impulsive aggression [117, 118].

The contributing role of steroid hormones is another focus of recent research. In a study of a multiple regression model including abuse/neglect history, psychopathy, and impulsivity, baseline cortisol explained 58% of the variance in trait aggression and 26% of the variance in state aggression. The study seems to support the hypothesis that abuse/neglect experiences predict a reduced hypothalamus-pituitary-adrenal (HPA)-axis reactivity, suggesting that a history of child maltreatment, psychopathy, and an impaired HPA-axis reactivity might act together to generate a confluence over aggressive behavior [118].

Recently, pathological appetitive aggression (to be distinguished from defensive one) has been hypothesized to result from an excessive activation of evolutionary conserved reward circuits, also mediating the rewarding effects of addictive drugs [119]. The authors suggest that inappropriate appetitive aggression shares core features with addiction: aggression is often sought despite adverse consequences, and relapse rates among aggressive offenders are as high as relapse rates in drug addiction.

Several genes have been investigated to explore a possible correlation with aggressive behavior. The most intensively studied is the catechol-O-methyltransferase (COMT) gene allocated on chromosome 22. COMT is involved in the metabolism of dopamine, a key neurotransmitter in schizophrenia pathophysiology. A meta-analysis involving 2370 individuals showed that male patients with schizophrenia who carried the low-activity methionine allele in the COMT gene had an increase in aggression risk by approximately 50% compared with homozygous valine patients [120]. A more recent Swedish cohort study was in line with the hypothesis that COMT genotypes modify the sensitivity to environment that confers either risk (methionine allele) or protection (valine allele) for aggressive behavior [121].

Recent data, evaluating the history of ExD across time, suggest a possible genetic disorder leading to dysregulated central dopamine transporter function that may be a precipitating cause of the condition of excitement and aggressiveness, as well as sudden death because of loss of autonomic function, progressing to cardiorespiratory collapse [122].

## Discussion

In this review, we have examined, within a quite complex and controversial literature, the phenomenon of aggression in European hospital settings. Our findings indicate the existence of some differences in rates of aggressive episodes among European countries. This can be due to organizational and environmental differences, such as the extension of teamwork or staff isolation and the characteristics of services and patient/staff ratios, or to differences in reporting aggression, rather than to real clinical differences.

In general, there is an agreement to consider that the interplay of different variables intervenes in determining the risk of aggressive behavior in psychiatric patients. The relationship between mental illness and aggression appears mediated by a combination of clinical, individual, and contextual risk factors, with drug abuse as a trans-dimensional variable which deserves particular attention and with an early age onset of drug abuse as an even higher risk of later occurrence of physically aggressive behavior. This could be explained with the impact of drugs on the neurobiological balance during adolescence, a crucial period for brain maturation.

Among sociodemographic variables, younger age appears independently correlated to an increased risk of aggression, suggesting a reduced capacity of emotional regulation, due either to the young age itself or to a higher acuity of symptom profile at the onset of psychosis [22].

The association between a history of childhood abuse and neglect and an increased risk of aggressive behavior in psychiatric patients has been highlighted in several European studies, confirming that the “cycle of violence” displays a transgenerational pattern.

It has also been pointed out how ward relational factors can often precipitate aggressive incidents. Furthermore, it has been hypothesized that ward architectural inadequacies that limit the possibility of patients to seek privacy, regulate relationships, and reduce stressors such as noise and arguments play a role in increasing levels of aggressiveness in hospitals.

In addition, research suggests a possible existence of neurobiological correlates of aggression in psychiatry.

Literature data show that the consequences of aggression are wide reaching, both for victims and actors. Victims of physical aggression—leaving aside physical damage—can report a vast array of psychological consequences, whose seriousness ranges from adjustment symptoms up to post-traumatic stress disorders [123•, 124]. Verbal aggression has been found to produce angst, depression, and other emotional distress, with repercussions on morale, professional replacement, quality of care, and patient recovery processes [125–127].

It has been hypothesized that patients with severe mental illness may lack skills to resolve interpersonal conflicts and consequently resort to physical violence [84]. If this hypothesis was confirmed, effective social skills training interventions could reduce aggressive enactments as well as victimization experiences.

On these premises, greater importance should be given to initiatives aimed at improving prevention, early recognition, and management of aggression in general hospital.

Recently, an updated version of the National Institute for Health and Clinical Excellence (NICE) guideline for short-term management in psychiatric settings has been developed by a multidisciplinary team of healthcare professionals, patients with a personal experience of aggressive behavior, their caregivers, and guideline methodologists [43••].

However, despite the growth in legislation and in the number of Health and Safety Authorities in various European member states [128], a lack of unifying procedures and standards emerges pertaining to ward safety and security in psychiatric and general hospitals [129].

## Conclusions

This review has some limitations. The first arises from the research design itself. Though this work explored different factors in the area, the studies included are heterogeneous and this review did not intend to categorize them depending on their design, methods, and primary outcomes; therefore, no definitive conclusion about associations between factors and risk of aggression can be made. Another limitation partially derives from the nature of both the literature available (which did not allow a more precise differentiation among countries, inpatients and medical environments) and the topic (which does not lend itself to more rigorous research approaches, such as randomized controlled trials).

Nonetheless, we believe that this review can be a valuable contribution to a better delineation of the phenomenon of aggression in European inpatient hospital settings. It also highlights the need to take a commitment both in using the predictive variables to create guidelines and procedures for prevention of aggressive behavior and in improving strategies to manage aggression in the general hospital.
